# Structural disconnection-based prediction of poststroke depression

**DOI:** 10.1038/s41398-022-02223-2

**Published:** 2022-11-03

**Authors:** Chensheng Pan, Guo Li, Ping Jing, Guohua Chen, Wenzhe Sun, Jinfeng Miao, Yanyan Wang, Yan Lan, Xiuli Qiu, Xin Zhao, Junhua Mei, Shanshan Huang, Lifei Lian, He Wang, Zhou Zhu, Suiqiang Zhu

**Affiliations:** 1 Department of Neurology, Tongji Hospital, Tongji Medical College, Huazhong University of Science and Technology, 430030 Wuhan, Hubei China; 2 Department of Neurology, Wuhan Central Hospital, 430014 Wuhan, Hubei China; 3 Department of Neurology, Wuhan First Hospital, 430022 Wuhan, Hubei China

**Keywords:** Predictive markers, Human behaviour, Depression

## Abstract

Poststroke depression (PSD) is a common complication of stroke. Brain network disruptions caused by stroke are potential biological determinants of PSD but their conclusive roles are unavailable. Our study aimed to identify the strategic structural disconnection (SDC) pattern for PSD at three months poststroke and assess the predictive value of SDC information. Our prospective cohort of 697 first-ever acute ischemic stroke patients were recruited from three hospitals in central China. Sociodemographic, clinical, psychological and neuroimaging data were collected at baseline and depression status was assessed at three months poststroke. Voxel-based disconnection-symptom mapping found that SDCs involving bilateral temporal white matter and posterior corpus callosum, as well as white matter next to bilateral prefrontal cortex and posterior parietal cortex, were associated with PSD. This PSD-specific SDC pattern was used to derive SDC scores for all participants. SDC score was an independent predictor of PSD after adjusting for all imaging and clinical-sociodemographic-psychological covariates (odds ratio, 1.25; 95% confidence interval, 1.07, 1.48; *P* = 0.006). Split-half replication showed the stability and generalizability of above results. When added to the clinical-sociodemographic-psychological prediction model, SDC score significantly improved the model performance and ranked the highest in terms of predictor importance. In conclusion, a strategic SDC pattern involving multiple lobes bilaterally is identified for PSD at 3 months poststroke. The SDC score is an independent predictor of PSD and may improve the predictive performance of the clinical-sociodemographic-psychological prediction model, providing new evidence for the brain-behavior mechanism and biopsychosocial theory of PSD.

## Introduction

Poststroke depression (PSD) is a common complication of stroke, affecting ~29% of patients at any time within 5 years poststroke [1]. PSD is correlated with poorer functional outcome and higher long-term mortality in stroke survivors [2]. For early prediction and intervention of PSD, risk factors including physical disability, stroke severity, history of depression, and cognitive impairment have been well-recognized [2]. However, it is unclear whether PSD is merely a “reactive” psychological response or whether there are biological factors related to brain damage that directly contribute to the development of PSD [3]. The association between lesion location and PSD, though extensively investigated as a potential biological factor of PSD, is still of considerable debate [2]. Neither early region-of-interest(ROI)-based lesion analyses nor latest voxel-based lesion-symptom mapping (VLSM) studies could provide consistent results for the neural substrates of PSD based on lesion location [4–6]. It is conceivable that PSD, like other depressive disorders, can be a complex disconnection syndrome and may not be mapped to a single brain region [5, 7]. The spatial topography information used in VLSM only represents a surface-level depiction of the lesion largely blind to its impact on the underlying brain network [5, 8, 9]. Novel techniques have been emerging for studying the association between lesion-induced functional or structural disconnection (FDC or SDC, respectively) and neuropsychiatric symptoms after focal brain damage [9]. Lesion network mapping (LNM) can indirectly estimate the FDC caused by each lesion using normative functional neuroimaging data and then statistically map the symptom of interest to a functional network [9, 10]. Similarly, another technique named disconnectome can indirectly measure the lesion-induced white matter SDC based on normative connectome atlas and then test the association between SDC and symptoms at tract or voxel level [11, 12]. Both approaches, without need for specialized neuroimaging, may prove broadly applicable and versatile for understanding the neural basis of neuropsychiatric symptoms after stroke. A widespread functional depression circuit centered on the left dorsolateral prefrontal cortex (DLPFC) has been identified in patients with focal brain damage using LNM and the lesion’s overlap status with this network may predict the risk and severity of PSD [5]. Recent evidence suggested that functional network disruption caused by stroke could be explained by SDC [13]. SDC might be a more upstream mechanism than functional network disruption in the pathogenesis of PSD. Moreover, the indirect SDC measures from disconnectome analysis were found superior to indirect FDC measures in predicting multiple behavioral deficits after stroke [11]. Here, we hypothesized that a widespread SDC pattern associated with PSD could be identified using disconnectome analysis and that the biological effect of SDC would be independently predictive of PSD after controlling for clinical-sociodemographic-psychological factors.

This study aimed to (1) identify the strategic SDC pattern for PSD at three months poststroke; (2) assess the value of SDC information in PSD prediction. This work may shed more light on the brain-behavior mechanism and explore new predictive biomarkers for PSD.

## Methods

### Participants and study design

A prospective cohort was enrolled from three independent hospitals in Wuhan City, Hubei Province, China, between May 2018 and August 2019. Institutional review boards reviewed and approved all study protocols. Written informed consent was obtained from all participants. All participants fulfilled the following inclusion criteria: (1) acute stroke confirmed with magnetic resonance imaging (MRI) or computed tomography (CT), with symptom onset to hospital admission <7 days, (2) age ≥18 years old. The exclusion criteria were: (1) brain dysfunction caused by non-vascular causes, such as brain tumors and traumatic brain injury, (2) history of depression, dementia and other psychiatric disorders, (3) communication problems due to aphasia, severe dysarthria, disturbance of understanding or consciousness, (4) unable to complete the follow-up, (5) transient ischemic attack and subarachnoid hemorrhage, (6) other concomitant neurological disorders, such as Parkinson’s disease and epilepsy, and (7) prior stroke history. For the 961 consecutive patients enrolled with the above criteria, baseline sociodemographic, clinical, psychological and radiographic information was collected within the first 48 hours after admission including: age, sex, education years, social support (social support rating scale, SSRS), living alone or not, premorbid physical exercise habit, prior stressful life event, acute ischemic stroke (AIS) subtype (Trial of Org 10172 in Acute Stroke Treatment, TOAST), stroke severity (National Institutes of Health Stroke Scale, NIHSS), cognitive impairment (Montreal Cognitive Assessment, MoCA), level of disability (Barthel Index, BI), acute management (tissue plasminogen activator, endovascular intervention), chronic comorbidities, personality (Eysenck Personality Questionnaire, which consists of three dimensions: extraversion, neuroticism and psychoticism), lesion localization, and the timing of imaging since stroke onset. Then 891 patients were assessed for presence of PSD at three months (90 ± 7 days) poststroke in the clinic. Information on antidepressant use and recent stressful life event was also collected. During data analysis, we further excluded patients with hemorrhagic stroke or without qualified neuroimages for lesion delineation. Finally, a total of 697 first-ever AIS patients with complete baseline and follow-up data were included in this study. Details are shown in Supplemental Fig. I.

### Behavioral assessment

The 17-item Hamilton Depression Rating Scale (HDRS) was applied to assess depression status at 3 months (90 ± 7 days) poststroke by two experienced raters with high interrater reliability (intraclass correlation coefficient: 0.917; 95% confidence interval: 0.790–0.967). With the diagnostic criteria in The Diagnostic and Statistical Manual of Mental Disorders, Fifth Edition (DSM-5) being met, HDRS ≥ 10 was regarded as presence of PSD [14].

### Image acquisition and preprocessing

Clinical neuroimages (MRI) performed at admission were collected for all 697 participants. Acquisition parameters were listed in Supplemental Table I. The lesions were manually segmented on MRI by an experienced rater (Chensheng Pan) blinded to PSD outcome in ITK-SNAP version 3.8.0 (www.itksnap.org). The lesion masks were supervised by another well-trained neurologist (Wenzhe Sun) for agreement. Spatial normalization to Montreal Neurological Institute (MNI 152) template was performed for original MRI images and native lesion masks with Clinical Toolbox [15] of Statistical Parametric Mapping (SPM12, Wellcome Trust Centre for Neuroimaging, London, United Kingdom) running on MATLAB R2021a (The MathWorks, Inc, Natick, MA). Visual inspection of the normalized lesion maps, as well as manual correction if necessary, were performed (Chensheng Pan and Wenzhe Sun). The lesion volume (cm^3^) adjusted for total brain volume was derived from the normalized lesion map.

### Statistical analysis

Baseline characteristics were compared between PSD and non-PSD. Continuous data were compared using nonparametric rank sum tests. The *χ*^2^ tests were used for comparison of categorical variables. Statistical significance was set to *P* < 0.05. All statistical analyses were performed in R software version 4.1.0 (The R Foundation for Statistical Computing, Vienna, Austria; www.r-project.org).

### Lesion analyses

The prevalence of PSD in patients with left vs right hemispheric strokes were compared using chi-square test (bihemispheric strokes excluded). The lesion overlap map for all 697 patients and lesion probability maps of PSD vs non-PSD groups were created to characterize the study cohort. Lesion subtraction analysis was performed to provide a descriptive result for possible regions implicated in PSD. VLSM was performed in NiiStat [16] with PSD as a binary variable. Only voxels involving at least five patients were included to maintain statistical power [17, 18]. One-tailed Liebermeister tests [19] were used to test the association between lesion status of each voxel and presence of PSD. To control the false positive rate in multiple comparisons, voxel-level family wise error (FWE) correction was performed with 10,000 random permutations [19]. Results were thresholded at *P*(FWE) < 0.05 at voxel level, and a minimal cluster size was determined with 10,000 permutations (pre-set voxel-wise threshold p < 0.001) [20]. For identification of significant clusters, the resulting Z score map was overlaid onto the “jhu” atlas in MRIcron v1.0.

### Voxel-based disconnection-symptom mapping

The SDC caused by each lesion was derived using Lesion Quantification Toolkit (LQT) [12] which incorporates a large-scale normative connectome atlas with novel algorithms for estimating lesion-induced SDC. With the lesion embedded into the full set of streamlines in the Human Connectome Project-842 (HCP-842) atlas [21], LQT output the voxel-wise disconnection severity map where voxel intensities corresponded to the percentage of all streamlines within each voxel that were expected to be disconnected by the lesion. All 697 disconnection severity maps were binarized at a severity threshold≥10% to exclude voxels insufficiently affected [12]. Binary disconnection maps of all 697 patients were overlapped on template to show the disconnection prevalence across white matter. The procedure of Voxel-based disconnection-symptom mapping (VDSM) were the same as in VLSM except for two points: (1) Only voxels disconnected in at least 5% patients (i.e. at least 35 patients when N = 697, supplementary VDSM analyses were performed with this threshold set at 5 patients or 70 patients) were included in VDSM; (2) replacing lesion map with binary SDC map as input. The resulting significant clusters from VDSM were embedded in HCP-842 atlas to identify the corresponding fiber tracts. Although our primary results were based on SDC map binarized at 10%, VDSM was additionally repeated with disconnection severity map binarized at lower (1%) and higher (20%) thresholds to examine whether the results would be significantly biased by the choice of threshold to define a disconnected voxel.

### Tract-wise disconnectome analysis

LQT also output tract-wise disconnection severity results for the 70 tracts defined in HCP-842 tractography atlas [12]. Disconnection severities for each of 70 tracts were compared between PSD and non-PSD using nonparametric rank sum tests with false discovery rate correction for multiple comparisons (corrected *P* < 0.05).

### SDC score and regression analyses

The SDC score was calculated as the weighted sum of intensities (Z scores) for those VDSM-significant voxels that overlapped with a patient’s non-binarized disconnection severity map (voxel-wise disconnection severities as weights) using fslmaths and fslstats in FMRIB Software Library (FSL) version 6.0. We performed univariable (Model 1) and multivariable (Models 2–3) logistic regression analyses to evaluate the association between SDC score (log-transformed for ease of analysis) and PSD in the entire sample. In the multivariable analysis, we constructed two models: Model 2, adjusted for neuroimaging covariates (lesion volume, lesion localization and timing of imaging since stroke onset); Model 3, additionally adjusted for all sociodemographic, clinical and psychological covariates. Variance inflation factors (VIFs) were calculated for the fully-adjusted model (Model 3) to check for multicollinearity among variables. We also tested the association between lesion volume and PSD by adjusting for lesion localization, timing of imaging and all non-imaging covariates.

### Split-half validation and predictive value of SDC score

Following preexisting literature [5, 22], we performed split-half analysis (i.e. 2-fold cross-validation) to test the generalizability of our results. The entire sample was randomly split into two halves (dataset 1: *n* = 349; dataset 2: *n* = 348). VDSM was repeated for each of the two datasets. For each patient in one dataset, SDC score was calculated based on VDSM results from the other dataset. Then SDC scores were compared between PSD and non-PSD groups. The entire sample was divided into three risk groups (low/medium/high) defined by SDC score tertiles. The true PSD prevalence and severities were compared among three risk groups using 2-by-3 *χ*^2^ test and nonparametric rank sum test, respectively. The above multivariable logistic regression analyses for SDC score were repeated in both dataset 1 and dataset 2.

To examine the added value of SDC score to well-known clinical-demographic and psychosocial predictors in PSD prediction, we first trained two logistic regression models in dataset 1: (1) we pre-selected 8 frequently-reported predictors in current literature [2, 23, 24] (stroke severity, level of disability, cognitive impairment, age, sex, education level, neuroticism and extroversion) to build a basic model; (2) SDC score was incorporated into the basic model to train an enhanced model. Both models were tested in dataset 2. All above modeling procedures were repeated again with dataset 1 and dataset 2 interchanged. The event-per-variable (EPVs) were controlled >10 for all models to ensured model stability. VIFs were calculated for all models to check for multicollinearity among predictors. The discrimination of each model was measured by area under receiver operating characteristic curve (AUC). The incremental improvement of adding SDC score to basic prediction model was measured by category-free net reclassification improvement (NRI) [25] and integrated discrimination improvement (IDI) [26] using R software (“PredictABEL” package). For two enhanced models with SDC score, we applied dominance analysis [27] to rank the relative importance of predictors using R software (“dominanceanalysis” package, fit function: r2.m), and then averaged the two results for visualization.

### VDSM at individual symptom level

Recent evidence suggests that the association between biopsychosocial factors and depression may be symptom-specific [28–31]. Unraveling mechanisms and risk factors of depression at individual symptom/dimension level has become a new research paradigm which is being advocated in the field of PSD [3, 5]. We performed exploratory VDSM analyses for each of 14 symptoms extracted from HDRS (12 depressive and 2 anxiety symptoms, see Supplemental Table II).

## Results

### Analyses of baseline parameters

Baseline parameters were compared in Table 1 between PSD and non-PSD. The 697 AIS patients were mildly affected with a median initial NIHSS of 3, of which 194 (27.8%) were rated as PSD at 3 months poststroke. The PSD group had significantly more females (*P* < 0.001), lower education level (*P* < 0.001), less premorbid physical exercise (*P* < 0.001), higher stroke severity (*P* < 0.001), higher levels of disability (*P* < 0.001), poorer cognitive function (*P* < 0.001), lower extraversion (*P* = 0.005), higher neuroticism (*P* < 0.001), and larger lesion size (*P* < 0.001). The AIS subtype composition also differed significantly between PSD and non-PSD (*P* = 0.033). Inter-group difference in lesion localization was not observed (*P* = 0.183). Antidepressant use was recorded in 76 (10.9%) patients during follow-up, among which 39 (51.3%) patients were still rated as PSD at 3 months poststroke. No new stressful life event was reported during follow-up.

**Table 1:** Baseline characteristics of the study sample.

Variable	Total	PSD	Non-PSD	*P* value
N	697	194	503	–
Sociodemographic factors				
Age, years	59 (52,66)	59.5 (52,66)	59 (52,66)	0.925
Sex, female	144 (20.7)	56 (28.9)	88 (17.5)	<0.001*
Education years	9 (9,12)	9 (6,12)	9 (9,12)	<0.001*
Social support level	38 (32,43)	38 (32,44)	38 (34,43)	0.841
Living alone	52 (7.5)	15 (7.7)	37 (7.4)	0.866
Premorbid physical exercise habit	231 (33.1)	43 (22.2)	188 (37.4)	<0.001*
Prior stressful life event	40 (5.7)	13 (6.7)	27 (5.4)	0.498
Clinical factors				
Stroke subtype				0.033*
large-artery atherosclerosis	464 (66.6)	139 (71.6)	325 (64.6)	–
cardioembolism	46 (6.6)	18 (9.3)	28 (5.6)	–
small-vessel occlusion	59 (8.5)	11 (5.7)	48 (9.5)	–
stroke of other determined etiology	42 (6)	7 (3.6)	35 (7)	–
stroke of undetermined etiology	86 (12.3)	19 (9.8)	67 (13.3)	–
NIHSS	3 (1,5)	4 (2,8)	2 (1,5)	<0.001*
BI	95 (60,100)	67.5 (40,100)	95 (65,100)	<0.001*
MoCA	20 (16,24)	17 (13,21)	21 (17,24)	<0.001*
Tissue plasminogen activator administered	46 (6.6)	12 (6.2)	34 (6.8)	0.784
Endovascular intervention	36 (5.2)	15 (7.7)	21 (4.2)	0.057
Diabetes mellitus	179 (25.7)	55 (28.4)	124 (24.7)	0.317
Hypertension	401 (57.5)	111 (57.2)	290 (57.7)	0.917
Hyperlipidaemia	147 (21.1)	37 (19.1)	110 (21.9)	0.417
Coronary heart disease	48 (6.9)	18 (9.3)	30 (6)	0.121
Psychological factors				
Neuroticism	9 (6,11)	9 (7,12)	8 (5,10)	<0.001*
Extroversion	11 (8,14)	11 (7,13)	11 (9,15)	0.005*
Psychoticism	5 (3,6)	5 (3,6)	5 (3,6)	0.874
Neuroimaging factors				
Timing of imaging since stroke onset, days	3 (2,3)	3 (1,4)	3 (2,3)	0.749
Lesion localization	–	–	–	0.183
left hemispheric	244 (35)	67 (34.5)	177 (35.2)	–
right hemispheric	274 (39.3)	85 (43.8)	189 (37.6)	–
bihemispheric	30 (4.3)	10 (5.2)	20 (4)	–
infratentorial	149 (21.4)	32 (16.5)	117 (23.3)	–
Lesion volume, cm^3^	5.0 (2.1,17.2)	9.5 (3.7,27.6)	4.1 (1.9,14.1)	<0.001*

Continuous variables are presented as median (interquartile range) and categorical variables as *n* (%).

*PSD* poststroke depression, *NIHSS* National Institutes of Health Stroke Scale, *BI* Barthel index, *MoCA* Montreal Cognitive Assessment.

*P < 0.05.

### Lesion analyses

**Fig. 1:**
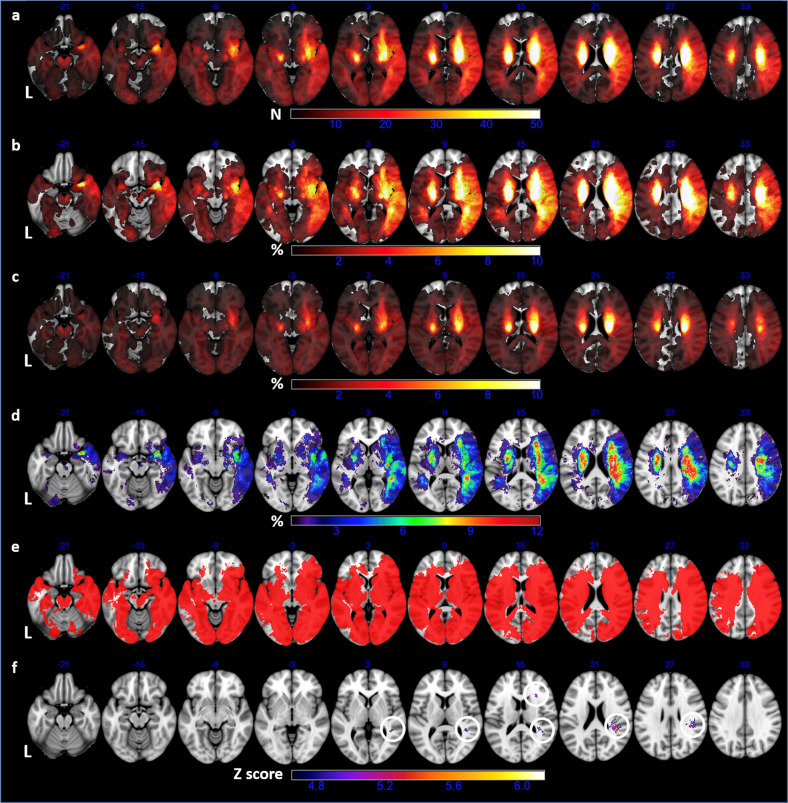
Results of Lesion Analyses. **a** overlap of all 697 lesion masks showing lesion frequency of the entire sample; **b** lesion prevalence(%) in PSD group; **c** lesion prevalence(%) in non-PSD group; **d** lesion subtraction plot (**b** minus **c**); **e** only voxels involving at least 5 patients were included in VLSM (red); **f** significant clusters (circled) from VLSM that survived FWE-corrected Z threshold of 4.65 and cluster size threshold of 22. Axial coordinates refer to MNI space in mm. L indicates left.

The prevalence of PSD was not significantly different between left vs right hemispheric strokes (27.5% vs 31.0%, *P* = 0.374). All other results from lesion analyses were shown in Fig. 1. The lesion frequency (1a) and coverage (1e) in left hemisphere were lower than in right hemisphere largely due to exclusion of phasic patients with lesions in the dominant hemisphere. We reached relatively high coverage (57.0%) of whole brain voxels in VLSM though bilateral frontal lobes and infratentorial structures were insufficiently covered (1e). The significant clusters from VLSM, involving multiple structures in right-sided frontal, parietal and temporal lobes, aligned with the hot zones in lesion subtraction plot (1d and 1 f). Detailed locations for these significant clusters were listed in Supplemental Table III.

### Voxel-based and tract-wise disconnectome analyses for PSD as a whole syndrome

**Fig. 2:**
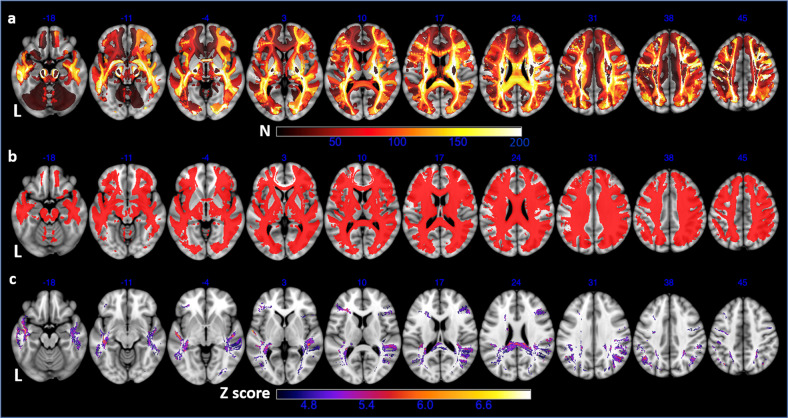
Results of the primary VDSM analysis in the entire sample. **a** overlap of 697 binary SDC maps showing SDC frequency across white matter; **b** only voxels disconnected in at least 5% of patients (i.e. 35 patients) were included in VDSM; **c** significant clusters from VDSM that survived FWE-corrected Z threshold of 4.47 and cluster size threshold of 60. Axial coordinates refer to MNI space in mm. L indicates left.

The primary VDSM results were shown in Fig. 2. The left frontal white matter was less frequently disconnected than the right counterpart largely due to exclusion of aphasic patients (2a). Significant voxels survived the voxel-level FWE correction and the cluster size threshold were mainly observed in bilateral temporal white matter and posterior corpus callosum, as well as white matter next to bilateral prefrontal cortex and posterior parietal cortex (2c). The significant clusters in VDSM were highly consistent across varied disconnection-frequency thresholds (5, 35, or 70 patients; Supplemental Fig. II). The fiber tracts intersected by these significant clusters, as well as significant results from tract-wise disconnectome analysis, were listed in Supplemental Table IV. Results from additional VDSM analyses with other binarization thresholds were shown in Supplemental Fig. III.

### SDC score and regression analyses

The results of univariable and multivariable analyses for the relationship between SDC score and PSD were shown in Table 2. In the fully-adjusted model controlling for various imaging and non-imaging covariates (Model 3), SDC score was independently predictive of PSD (odds ratio, OR 1.25; 95% confidence interval, CI 1.07,1.48; *P* = 0.006). Multicollinearity was not detected in Model 3 (all VIFs < 3). Though lesion volume was significantly larger in PSD than in non-PSD (*P* < 0.001, Table 1), it was not associated with PSD after controlling for lesion localization, timing of imaging, and non-imaging covariates (OR 1.01; 95% CI 0.99,1.02; *P* = 0.200).

**Table 2:** Univariable and multivariable logistic regression analyses for the association between SDC score and PSD.

Variable	Model 1		Model 2		Model 3	
	OR (95% CI)	*P* value	OR (95% CI)	*P* value	OR (95% CI)	*P* value
SDC score (log-transformed)	1.42 (1.24, 1.64)	<0.001*	1.39 (1.18, 1.66)	<0.001*	1.25 (1.07,1.48)	0.006*

Model 1: crude model;

Model 2: adjusted for neuroimaging covariates including timing of imaging since stroke onset, lesion localization and lesion volume;

Model 3: additionally adjusted for all sociodemographic, clinical, and psychological factors.

*SDC* structural disconnection, *OR* odds ratio, *CI* confidence interval.

*P < 0.05.

### Split-half validation and predictive value of SDC score

**Fig. 3:**
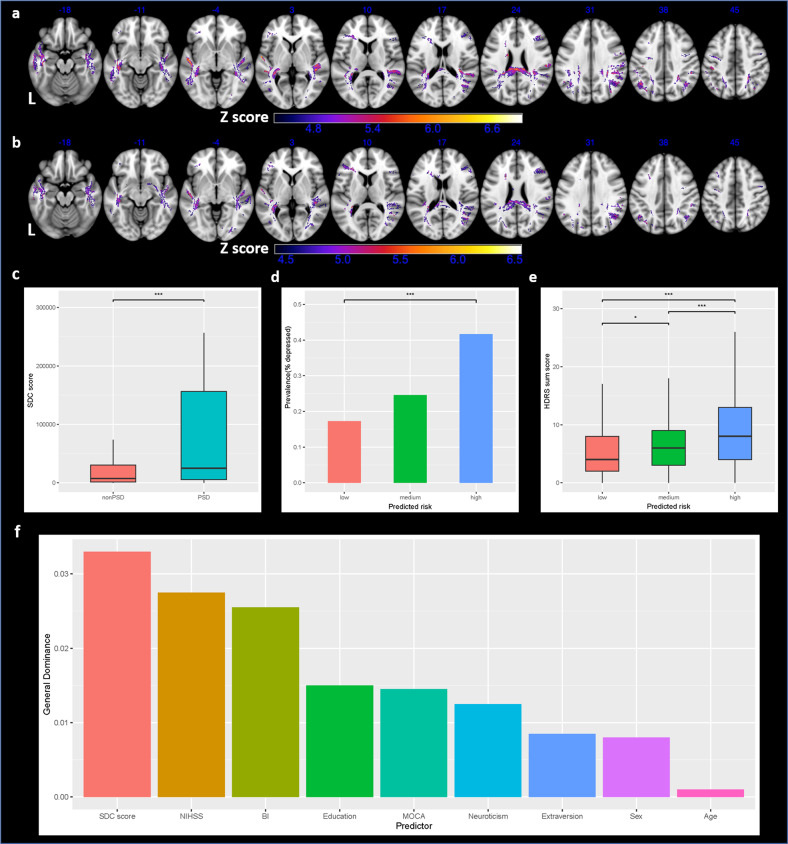
Results of split-half analysis. **a**, **b** VDSM results from dataset 1 and dataset 2, respectively; **c** SDC score compared between PSD and non-PSD; **d** true PSD prevalence compared among three risk groups defined by SDC score tertiles; **e** true PSD severities compared among three risk groups defined by SDC score tertiles; **f** averaged predictor importance of two enhanced models. Axial coordinates refer to MNI space in mm. L indicates left.

Results for split-half replication were shown in Fig. 3. VDSM results from dataset 1 (3a) and dataset 2 (3b) were highly similar. In this section, each patient’s SDC score was calculated based on VDSM results from the dataset he/she did not belong. SDC scores were significantly higher in PSD than in non-PSD (3c). SDC score alone could predict the risk and severity of PSD (3d-3e). SDC score was independently associated with PSD in both datasets after controlling all imaging and non-imaging covariates (mean odds ratio 1.20, both *P* < 0.05). In the two rounds of modeling, NRI and IDI suggested significant improvement of predictive performance in enhanced models (Table 3). The mean AUC for two basic models was 0.737 in training and 0.721 in testing, while the mean AUC for two enhanced models was 0.754 in training and 0.744 in testing. Multicollinearity was not detected (VIFs < 3 for all models). In dominance analyses for enhanced models, SDC score ranked the highest in terms of predictor importance (3f).

**Table 3:** Improvement of model performance after introduction of SDC score (enhanced model vs basic model) in split-half analyses.

Variable	Models trained in dataset 1 and tested in dataset 2				Models trained in dataset 2 and tested in dataset 1			
	Training set effect size (95% CI)	*P* value	Testing set effect size (95% CI)	*P* value	Training set effect size (95% CI)	*P* value	Testing set effect size (95% CI)	*P* value
NRI	0.339 (0.117, 0.562)	0.003*	0.305 (0.077,0.533)	0.009*	0.399 (0.166,0.632)	<0.001*	0.322 (0.093,0.550)	0.006*
IDI	0.042 (0.017, 0.066)	<0.001*	0.028 (0.003,0.053)	0.027*	0.023 (0.004,0.042)	0.017*	0.037 (0.020,0.054)	<0.001*

*NRI* category-free net reclassification improvement, *IDI* integrated discrimination improvement, *CI* confidence interval.

*P < 0.05.

### VDSM at individual symptom level

Five symptoms (*depressed mood, loss of interest, retardation, psychiatric anxiety, general somatic symptoms*) were more strongly correlated with SDC (Supplemental Figs. IV–VIII), while the other nine symptoms showed negative results.

## Discussion

To the best of our knowledge, this is first prospective cohort study introducing VDSM-defined SDC information into PSD prediction. We identified the SDC pattern associated with PSD at 3 months poststroke and proposed a new imaging biomarker that independently predicts PSD.

In our cohort, the incidence of PSD (27.8%) at three months poststroke is comparable with previous studies [1]. To explore the neural substrate of PSD, we first performed standard lesion analyses and then focused on the underlying structural connectome disruption caused by lesions. PSD is not associated with lesion laterality (left vs right hemisphere) in our sample. The VLSM results and lesion subtraction plot converge to the association between PSD and multiple brain regions in the right frontal, temporal and parietal lobes (Fig. 1). The results may not imply that PSD is more correlated with right hemisphere considering the relatively lower lesion frequency and therefore lower statistical power in left hemisphere. We compared our results with preexisting PSD publications using lesion-symptom mapping (either voxel-based or multivariate). Results are highly heterogeneous in studies focusing on lesion location (Supplemental Table V). Recent evidence suggests that depressive disorders may be better represented in a widespread brain network rather than any single brain region [5, 7]. For example, Michael D. Fox’s group found that lesions associated with depression after focal brain damage (ischemic stroke, hemorrhagic stroke and traumatic brain injury), as well as effective brain stimulation targets for major depressive disorder (MDD), could be mapped to a common functional network spanning multiple lobes bilaterally [5, 32].

Applying novel disconnectome technique and voxel-wise analysis, we identified the association between SDC of certain white matter regions and PSD (Fig. 2). First, SDC within bilateral temporal white matter may be associated with PSD. A prior study showed the similar result that lesions in temporal lobes were associated with PSD three months poststroke [33]. Reduced gray matter volume of superior and middle temporal gyri were observed in MDD [34]. Second, SDC next to bilateral limbic structures (hippocampi and amygdalae) and bilateral prefrontal cortex are associated with PSD in our study, which is in line with the fronto-limbic model described in MDD [35] and vascular depression [36]. The role of prefrontal cortex in depression has been confirmed by studies demonstrating that symptoms of PSD or vascular depression can be significantly alleviated after repetitive transcranial magnetic stimulation targeted at DLPFC [37, 38]. LNM analysis also identified a functional depression circuit centered on the DLPFC [5]. Fourth, we found the association between posterior corpus callosum disconnection and PSD, though the role of posterior corpus callosum disconnection in MDD had been described [39]. The posterior corpus callosum, connecting bilateral temporal, parietal and occipital cortices, may play a vital role in maintaining stable functional communication between hemispheres [40]. Fifth, significant voxels were observed next to bilateral posterior parietal cortex which is one of the major associative regions in human brain and integrates information from somatosensory, auditory, visual, motor, cingulate and prefrontal cortices [41]. The important role of frontoparietal network (composed of posterior parietal cortex and DLPFC) in depression had been described [42]. The fiber tracts heavily intersected by significant clusters from VDSM are largely consistent with significant tracts from tract-wise disconnectome analysis (Supplemental Table IV). These PSD-associated tracts involve association pathways (e.g. superior/middle/inferior longitudinal fasciculus, arcuate fasciculus, inferior fronto-occipital fasciculus), projection pathways (acoustic/optic radiation) and commissural pathways (corpus callosum) [21]. The loss of integrity for these inter-lobar and inter-hemispheric connections may be the key neural substrate of PSD. It’s worth noting that a single lesion can disconnect multiple fiber tracts intersecting with it, and disconnection of a fiber tract may result from lesions anywhere along its course. This might explain the inconsistency of results from studies focusing on lesion itself. Of note, similar to the LNM-defined functional depression circuit [5, 32], our PSD-associated SDC pattern also involves multiple lobes bilaterally. The association between structural and functional connectivity, or between SDC and FDC, is not fully understood and still warrants further investigation [13].

Additional VDSM analyses with SDC map binarized at different severity thresholds (1%, 20%) revealed high similarity between results (Supplemental Fig. III), suggesting that VDSM may not be strongly biased by the choice of threshold to define a disconnected voxel. Based on the primary VDSM results, we derived SDC scores for all 697 participants. SDC score remained as an independent predictor of PSD after controlling for lesion volume, lesion localization, timing of imaging, as well as all clinical-demographic and psychosocial factors (Table 2). Lesion volume was not independently associated with PSD, as observed previously [43]. VDSM results were stable and generalizable in split-half replication (Fig. 3a, b). We may use SDC score alone for PSD risk stratification (Fig. 3d, e). Considering the biopsychosocial multifactorial nature of PSD, SDC score can be integrated with clinical, sociodemographic and psychological predictors to build a multidimensional prediction model. The classification performance of the basic prediction models was significantly improved (significant NRI and IDI) when SDC score was added, indicating a promising role of SDC information in PSD prediction (Table 3). The relative importance of each predictor in enhanced models is measured with general dominance (average contribution) in dominance analysis. SDC score may outperform three well-known clinical predictors (stroke severity, level of disability, cognitive impairment) and all psychosocial factors in PSD prediction (Fig. 3f). This result is in accordance with the biopsychosocial theory that PSD is not merely a psychosocial response but also the direct consequence of neurobiological damage after stroke [2]. VDSM analyses at symptom level suggest that the association between SDC and PSD may be symptom-specific: depressed mood, retardation and general somatic symptoms were correlated with anterior (especially left frontal) SDC (Supplemental Figs. IV–VI); psychiatric anxiety was more strongly associated with posterior SDC (Supplemental Fig. VII); *loss of interest* was mapped to both anterior and posterior white matter (Supplemental Fig. VIII). The other 9 symptoms are less likely to be associated with SDC and may result from other mechanisms: (1) psychosocial response to disability; (2) other biological factors (e.g. inflammation); (3) symptom-symptom interactions [44–46]. Future studies may not only treat PSD as a whole syndrome but also explore risk factors or mechanisms for individual symptoms and aspects of PSD [3].

Our VDSM results tied well with brain network theory of depression and offered new insights on the neural substrate of PSD. The independent predictive effect and added value of SDC information in PSD prediction may provide more evidence for the biopsychosocial model of PSD. Our study, along with recent evidence showing that preserved structural connections may serve as an essential mediator between treatment and favorable clinical outcome in AIS patients [47], may inform new preventative and therapeutic strategies for PSD. A variety of approaches, enabling preservation of anatomical connection after central nervous tissue injury by promoting axonal regeneration, revascularization and neuronal survival [48], could be effective in reducing the risk and severity of PSD and improving functional outcome after stroke. However, some limitations still exist in our study. First, selection bias (e.g. exclusion of aphasic patients who tend to have higher NIHSS and more depressive symptoms, which is a common limitation in the field of PSD research) might limit the generalizability of our results. Applying depression scales applicable for aphasic patients [49] may be helpful. Second, we performed massive univariate tests with dichotomized behavioral data in VDSM. The mass-univariate approach is vulnerable to consistent errors resulting from collateral vasculature [50]. Multivariate techniques [51, 52] may serve as promising alternatives in future studies. However, lesion-symptom inference based on mass-univariate approach is still widely accepted [18, 53]. Third, disconnection map in our study was indirectly estimated from the HCP-842 atlas with the lesion embedded in, thus not necessarily representing the actual SDC. However, the HCP-842 atlas is the averaged result of large-scale normal population, thus well-suited for use as a reference for white matter anatomy [12]. LNM or disconnectome studies based on normative atlases are less costly and more generalizable [11]. The clear temporal order between lesion-induced disconnection and PSD outcome allows causal inference which can hardly be made in most direct functional neuroimaging studies (revealing correlation instead) [9]. The atlas-based approach can be complemented by other advanced methodology including direct connectome analysis [54]. Fourth, the baseline depression status was not included as a covariate. There is inter-individual variability at baseline that likely contributes to inter-individual variability at 3 months post-stroke that cannot be accounted for. Covarying for depression at baseline can maximize variance related to the stroke specifically. Finally, future studies in population of various regions or ethnicity are entailed in validating the role of SDC in the etiology of PSD.

In conclusion, a strategic SDC pattern involving multiple lobes bilaterally is identified for PSD at 3 months poststroke. The baseline SDC score is an independent predictor of PSD and may improve the predictive performance of the clinical-sociodemographic-psychological prediction model, providing new evidence for the brain-behavior mechanism and biopsychosocial theory of PSD.

## Supplementary information

Supplemental Material
