# Supplementary material for: Structural disconnection-based prediction of poststroke depression
Source: Transl Psychiatry. 2022 Nov 3;12:461. doi: 10.1038/s41398-022-02223-2 (PMC9633711; doi:10.1038/s41398-022-02223-2)
Supplement: Supplementary file 1 — Supplemental Material [file 41398_2022_2223_MOESM1_ESM.docx]

**Supplemental Material**

Supplemental Table I. Neuroimaging protocols

|  | MRI |
| --- | --- |
| Tongji Hospital | GE’s Discovery MR750 3.0T scanner;  Axial plane;  T1WI: TR=2992.3ms, TE=24ms, TI=859ms, slice thickness =5mm, intersection gap =1.5mm, matrix =320*320, FOV =240*240mm^2^;  T2WI: TR=4650ms, TE=102ms, slice thickness =5mm, intersection gap =1.5mm, matrix =320*224, FOV =240*240mm^2^;  T2 FLAIR: TR=8000ms, TE=160ms, TI=2100ms, slice thickness =5mm, intersection gap =1.5mm, matrix =256*256, FOV =240*240mm^2^;  DWI: TR=3000ms, TE=65ms, slice thickness =5mm, intersection gap =1.5mm, matrix =256*256, FOV =240*240mm^2^; ADC map was derived from b0 and b1000 images. |
| Wuhan Central Hospital | Siemens’ MAGNETOM Skyra 3.0T MRI scanner;  Axial plane;  T1WI: TR=2000ms, TE=11ms, TI=825ms, slice thickness =5mm, intersection gap =1.5mm, matrix =320*320, FOV =220*220mm^2^;  T2WI: TR=4000ms, TE=99ms, slice thickness =5mm, intersection gap =1.5mm, matrix =512*512, FOV =240*240mm^2^;  T2 FLAIR: TR=8000ms, TE=84ms, TI=2220ms, slice thickness =5mm, matrix =320*320, FOV =220*220mm^2^;  DWI: TR=4040ms, TE=64ms, slice thickness =5mm, intersection gap =1.5mm, matrix =160*160, FOV =220*220mm^2^; ADC map was derived from b0 and b1000 images. |
| Wuhan First Hospital | Siemens’ MAGNETOM Vida 3.0T MRI scanner;  Axial plane;  T1WI: TR=1800ms, TE=11ms, TI=818ms, slice thickness =5mm, intersection gap =1.5mm, matrix =320*320, FOV =230*230mm^2^;  T2WI: TR=4500ms, TE=107ms, slice thickness =5mm, intersection gap =1.5mm, matrix =384*384, FOV =230*230mm^2^;  T2 FLAIR: TR=7000ms, TE=87ms, TI=2220ms, slice thickness =5mm, intersection gap =1.5mm, matrix =320*320, FOV =230*230mm^2^;  DWI: TR=4040ms, TE=64ms, slice thickness =5mm, intersection gap =1.5mm, matrix =160*160, FOV =230*230mm^2^; ADC map was derived from b0 and b1000 images. |

T1WI indicates T1 weighted image; T2WI, T2 weighted image; T2 FLAIR, T2 fluid attenuated inverse recovery; DWI, diffusion weighted image; ADC, apparent diffusion coefficient; TR, repetition time; TE, echo time; TI, inversion time; FOV, field of view; MRI, magnetic resonance imaging.

Supplemental Table II. Symptoms extracted from HDRS for VDSM analyses

| **Symptom** | **Score range** | **Prevalence(%)*** |
| --- | --- | --- |
| **Depressive symptoms** |  |  |
| Depressed mood | 0-4 | 53.7 |
| Feelings of guilt | 0-4 | 39.1 |
| Suicide | 0-4 | 9.5 |
| Initial insomnia | 0-2 | 37.2 |
| Insomnia during the night | 0-2 | 39.1 |
| Delayed insomnia | 0-2 | 35.0 |
| Loss of interest | 0-4 | 36.4 |
| Retardation | 0-4 | 27.8 |
| Agitation | 0-4 | 34.8 |
| Gastrointestinal somatic symptoms | 0-2 | 24.1 |
| General somatic symptoms | 0-2 | 32.1 |
| Weight loss | 0-2 | 28.0 |
| **Anxiety symptoms** |  |  |
| Psychiatric anxiety | 0-4 | 57.6 |
| Somatic anxiety | 0-4 | 31.2 |

*symptom score≥1 was considered presence of the symptom.

Supplemental Table III. Significant voxels in VLSM per region of interest

| Region | Peak coordinate | | | N of voxels | Mean Z score |
| --- | --- | --- | --- | --- | --- |
|  | x | y | z |  |  |
| Right inferior frontal gyrus | 28 | 22 | 16 | 129 | 4.960 |
| Right supramarginal gyrus | 38 | -34 | 25 | 1319 | 4.972 |
| Right angular gyrus | 42 | -32 | 23 | 270 | 4.959 |
| Right posterior corona radiata | 29 | -27 | 23 | 123 | 4.880 |
| Right posterior thalamic radiation | 40 | -35 | 24 | 164 | 4.961 |
| Right superior longitudinal fasciculus | 37 | -35 | 25 | 460 | 4.944 |
| Right posterior insula | 31 | -19 | 19 | 26 | 4.860 |
| Right posterior superior temporal gyrus | 41 | -35 | 24 | 275 | 5.007 |
| Right posterior middle temporal gyrus | 42 | -37 | 23 | 244 | 4.882 |

The number of significant voxels in each region was calculated with VLSM results overlapped onto “jhu” atlas in MRIcron software (v1.0.20190902, 64-bit Windows).

Supplemental table IV. Fiber tracts implicated in PSD identified with **voxel-based vs tract-wise** disconnectome analyses

| % tract disconnection by significant clusters in VDSM (descending order) | Tract abbreviation | Tract full name |
| --- | --- | --- |
| 100 | **'MdLF_L'*** | Middle Longitudinal Fasciculus, left |
| 100 | **'MdLF_R'*** | Middle Longitudinal Fasciculus, right |
| 100 | **'OR_L'*** | Optic Radiation, left |
| 99.953918 | **'ILF_L'*** | Inferior Longitudinal Fasciculus, left |
| 99.097816 | **'IFOF_L'*** | Inferior Fronto-Occipital Fasciculus, left |
| 98.404251 | **'OR_R'*** | Optic Radiation, right |
| 96.172737 | **'CCPosterior'*** | Corpus Callosum, posterior |
| 95.545654 | 'OPT_L' | Occipitopontine Tract, left |
| 95.063469 | **'EMC_R'*** | Extreme Capsule, right |
| 93.701942 | **'IFOF_R'*** | Inferior Fronto-Occipital Fasciculus, right |
| 88.894577 | **'SLF_R'*** | Superior Longitudinal Fasciculus, right |
| 82.969437 | **'AF_R'*** | Arcuate Fasciculus, right |
| 78.634438 | **'AF_L'*** | Arcuate Fasciculus, left |
| 77.777779 | 'F_L' | Fornix, left |
| 77.586212 | 'TPT_L' | Temporopontine Tract, left |
| 75.612785 | **'AR_L'*** | Acoustic Radiation, left |
| 73.964218 | **'AC'*** | Anterior Commissure |
| 70.918365 | 'OPT_R' | Occipitopontine Tract, right |
| 66.771164 | **'ILF_R'*** | Inferior Longitudinal Fasciculus, right |
| 59.926922 | 'EMC_L' | Extreme Capsule, left |
| 57.323055 | 'CT_L' | Corticothalamic Pathway, left |
| 47.938145 | 'TPT_R' | Temporopontine Tract, right |
| 47.706421 | **'AR_R'*** | Acoustic Radiation, right |
| 47.096775 | 'VOF_R' | Vertical Occipital Fasciculus, right |
| 46.093113 | 'U_L' | U-fiber, left |
| 45.50214 | 'SLF_L' | Superior Longitudinal Fasciculus, left |
| 45.020668 | 'CCMidAnterior' | Corpus Callosum, mid-anterior |
| 39.369827 | 'CT_R' | Corticothalamic Pathway, right |
| 38.251583 | 'CST_L' | Corticospinal Tract, left |
| 37.6241 | 'CS_L' | Corticostriatal Pathway, left |
| 36.968578 | 'CCCentral' | Corpus Callosum, central |
| 32.975307 | 'U_R' | U-fiber, right |
| 27.572018 | 'FPT_L' | Frontopontine Tract, left |
| 26.26527 | 'PPT_L' | Parietopontine Tract, left |
| 23.132036 | 'UF_R' | Uncinate Fasciculus, right |
| 22.945686 | **'CCAnterior'*** | Corpus Callosum, anterior |
| 17.535971 | 'UF_L' | Uncinate Fasciculus, left |
| 15.168539 | 'CCMidPosterior' | Corpus Callosum, mid-posterior |
| 14.169571 | 'VOF_L' | Vertical Occipital Fasciculus, left |
| 13.449635 | 'FAT_R' | Frontal Aslant Tract, right |
| 13.114754 | 'FAT_L' | Frontal Aslant Tract, left |
| 10.204856 | 'CS_R' | Corticostriatal Pathway, right |
| 9.6109838 | 'C_R' | Cingulum, right |
| 9.2584658 | 'PPT_R' | Parietopontine Tract, right |
| 5.6084657 | 'C_L' | Cingulum, left |
| 1.2955465 | 'CST_R' | Corticospinal Tract, right |
| 0.067024134 | 'FPT_R' | Frontopontine Tract, right |
| 0 | 'CB_L' | Cerebellum, left |
| 0 | 'CB_R' | Cerebellum, right |
| 0 | 'CTT_L' | Central Tegmental Tract, left |
| 0 | 'CTT_R' | Central Tegmental Tract, right |
| 0 | 'DLF_L' | Dorsal Longitudinal Fasciculus, left |
| 0 | 'DLF_R' | Dorsal Longitudinal Fasciculus, right |
| 0 | 'F_R' | Fornix, right |
| 0 | 'ICP_L' | Inferior Cerebellar Peduncle, left |
| 0 | 'ICP_R' | Inferior Cerebellar Peduncle, right |
| 0 | 'LL_L' | Lateral Lemniscus, left |
| 0 | 'LL_R' | Lateral Lemniscus, right |
| 0 | 'MCP' | Middle Cerebellar Peduncle |
| 0 | 'MLF_L' | Medial Longitudinal Fasciculus, left |
| 0 | 'MLF_R' | Medial Longitudinal Fasciculus, right |
| 0 | 'ML_L' | Medial Lemniscus, left |
| 0 | 'ML_R' | Medial Lemniscus, right |
| 0 | 'PC' | Posterior Commissure |
| 0 | 'RST_L' | Rubrospinal Tract, left |
| 0 | 'RST_R' | Rubrospinal Tract, right |
| 0 | 'SCP' | Superior Cerebellar Peduncle |
| 0 | 'STT_L' | Spinothalamic Tract, left |
| 0 | 'STT_R' | Spinothalamic Tract, right |
| 0 | 'V' | Vermis |

* **statistically significant in tract-wise disconnectome analysis.**

The 70 tracts are defined in Human Connectome Project-842 tractography atlas by Yeh et al., 2018[1].

| **Study** | **Sample size** | **Timing of behavioral assessment since lesion onset** | **Behavioral assessment method** | **Behavioral outcome** | **Regions implicated** |
| --- | --- | --- | --- | --- | --- |
| **VLSM** |  |  |  |  |  |
| Gozzi et al., 2014[2] | 55 | Within 12 days and at 1-month poststroke | DSM-4 and HADS | Binary PSD diagnosis (DSM-4 met and HADS>11) | Negative |
| Kim et al., 2017[3] | 24 | Within 3 months | DSM-4 and GDS | Binary PSD diagnosis (DSM-4 met and GDS>16) | Posterior lobe of the left cerebellar hemisphere |
| Padmanabhan et al., 2019[4] | 461 | Varied by 5 datasets with lesions of different etiologies including stroke; ranges from 28 days to 39 years | Varied by datasets: Neuro-QOL, GDSS, PHQ-9, HADS plus MINI, BDI-II | Binary PSD diagnosis (threshold varied by datasets: Neuro-QOL≥59.9, GDSS≥11, PHQ-9≥10, HADS≥11 with DSM-4 net, BDI-II≥20) | Negative |
| Klingbeil et al, 2022[5] | 270 | At 6 months | HADS | Continuous sum score | Right putamen |
| This work, 2022 | 697 | At 3 months (90±7 days) | HDRS | Binary PSD diagnosis (DSM-5 met and HRSD≥10) | See Supplemental table III |
| **MLSM** |  |  |  |  |  |
| Grajny et al., 2016[6] | 39 | At least 6 months | SADQ | Continuous sum score | Left dorsolateral prefrontal cortex |
| Weaver et al., 2021[7] | 553 | Within 1 year, ranges from 1 to 361 days | GDS | Continuous sum score | Right amygdala and pallidum |

Supplemental table V. Inconsistent results from current lesion-symptom mapping studies for PSD

VLSM indicates voxel-based lesion-symptom mapping; MLSM, multivariate lesion-symptom mapping; DSM, Diagnostic Statistical Manual of Mental Disorders; HADS, Hospital Anxiety and Depression Scale; GDS, Geriatric Depression Scale; Neuro-QOL, Neuro-QOL Depression Scale; GDSS, Geriatric Depression Score Short Form; PHQ, Patient Health Questionnaire; MINI, Mini-International Neuropsychiatric Interview; BDI, Beck Depression Inventory; HDRS, Hamilton Depression Rating Scale; SADQ, Stroke Aphasic Depression Questionnaire.


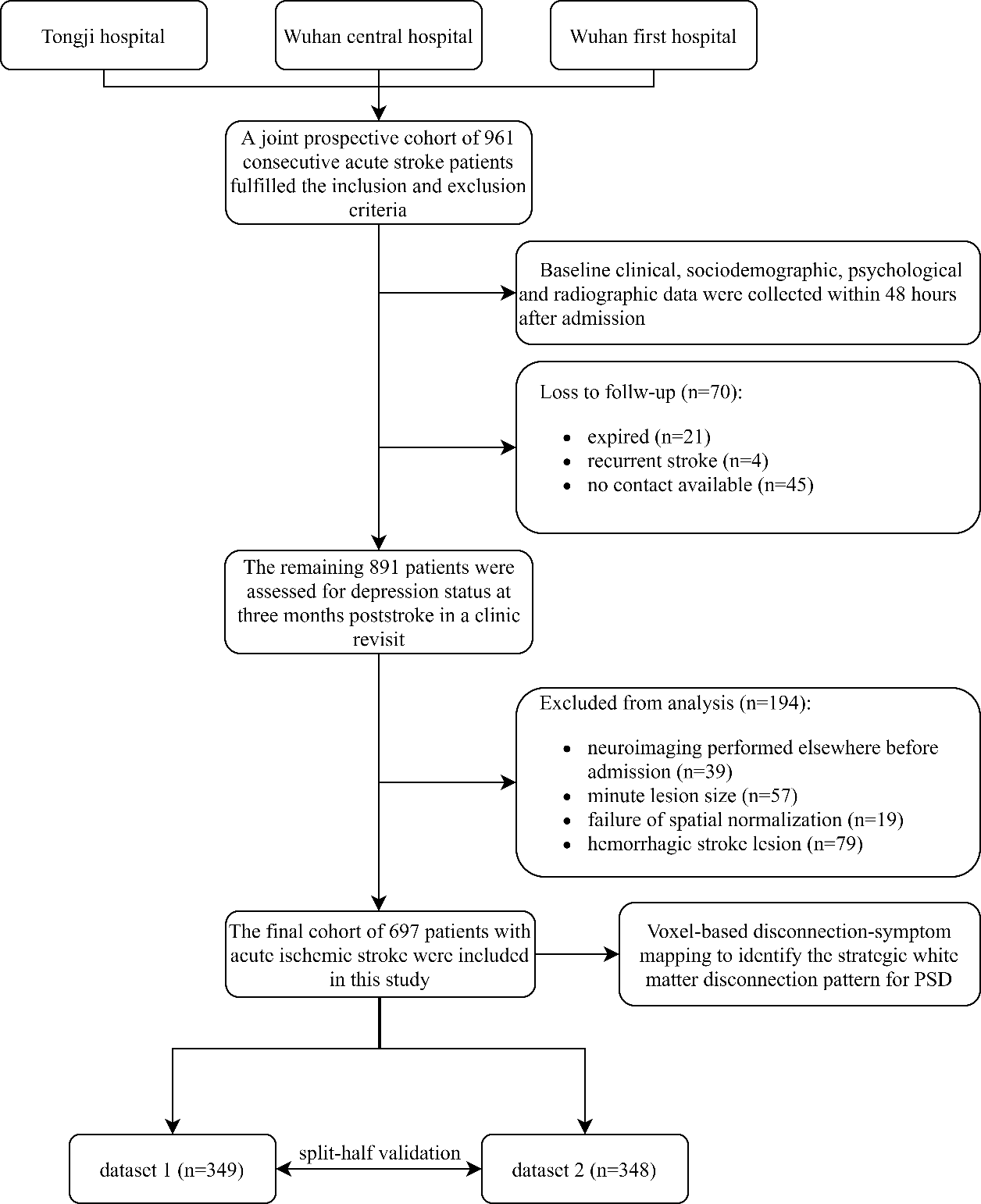


**Supplemental Figure I. Flowchart for patient selection and study design**


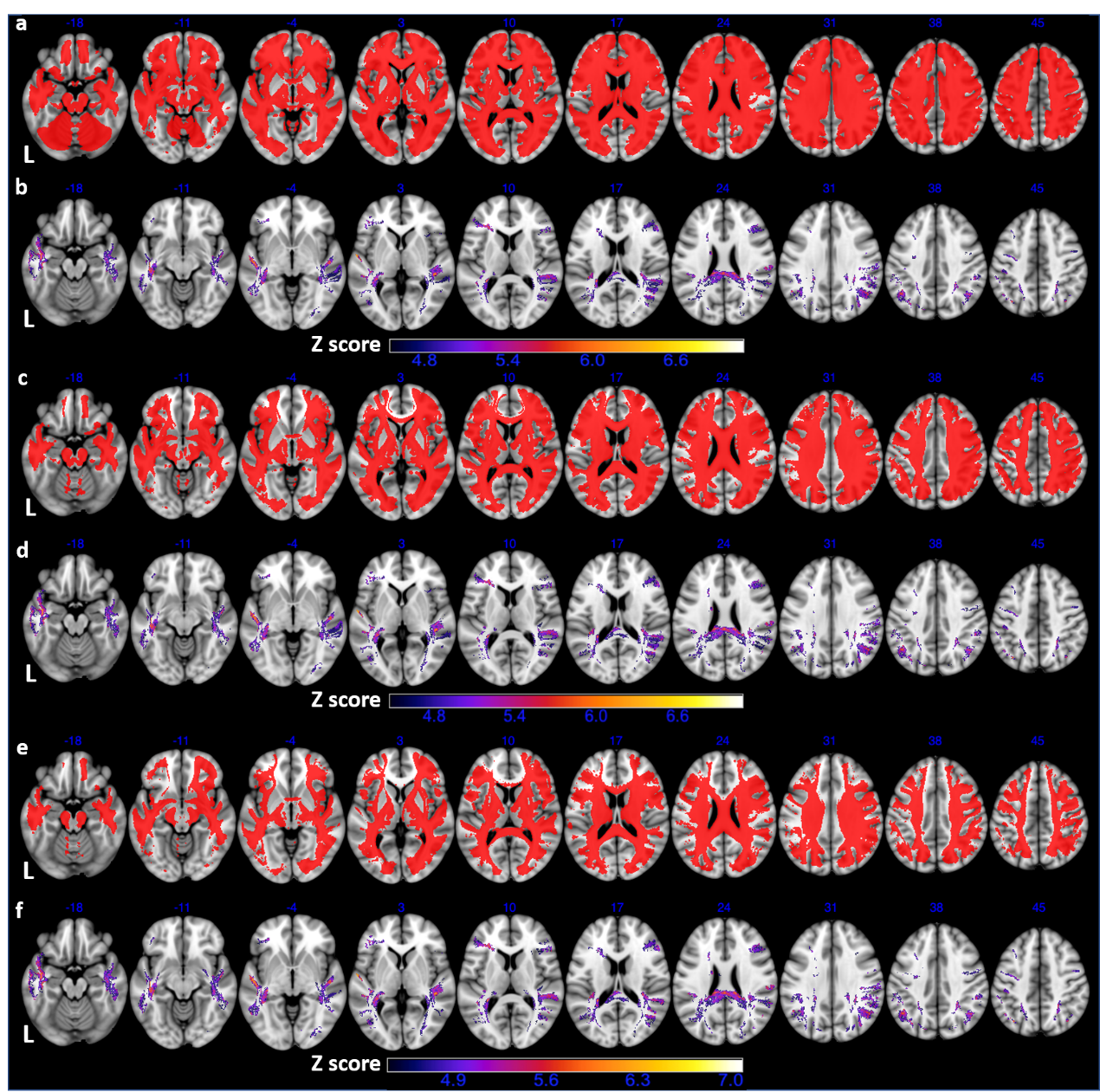


**Supplemental Figure II. disconnection coverage (a,c,e) and VDSM results (b,d,f) with varied disconnection-frequency thresholds.** Disconnection-frequency threshold was set at: 5 patients for a-b; 5% (i.e. 35 patients when N=697) for c-d; 10% (i.e. 70 patients when N=697) for e-f. A 10% total sample cutoff, though ideal in the field of lesion-symptom mapping, resulted in markedly less voxels to be included in VDSM (552 808 voxels were included in e, compared with 995 083 voxels in a and 742 805 voxels in c). However, the significant clusters in VDSM were highly consistent across varied disconnection-frequency thresholds (b,d,f).


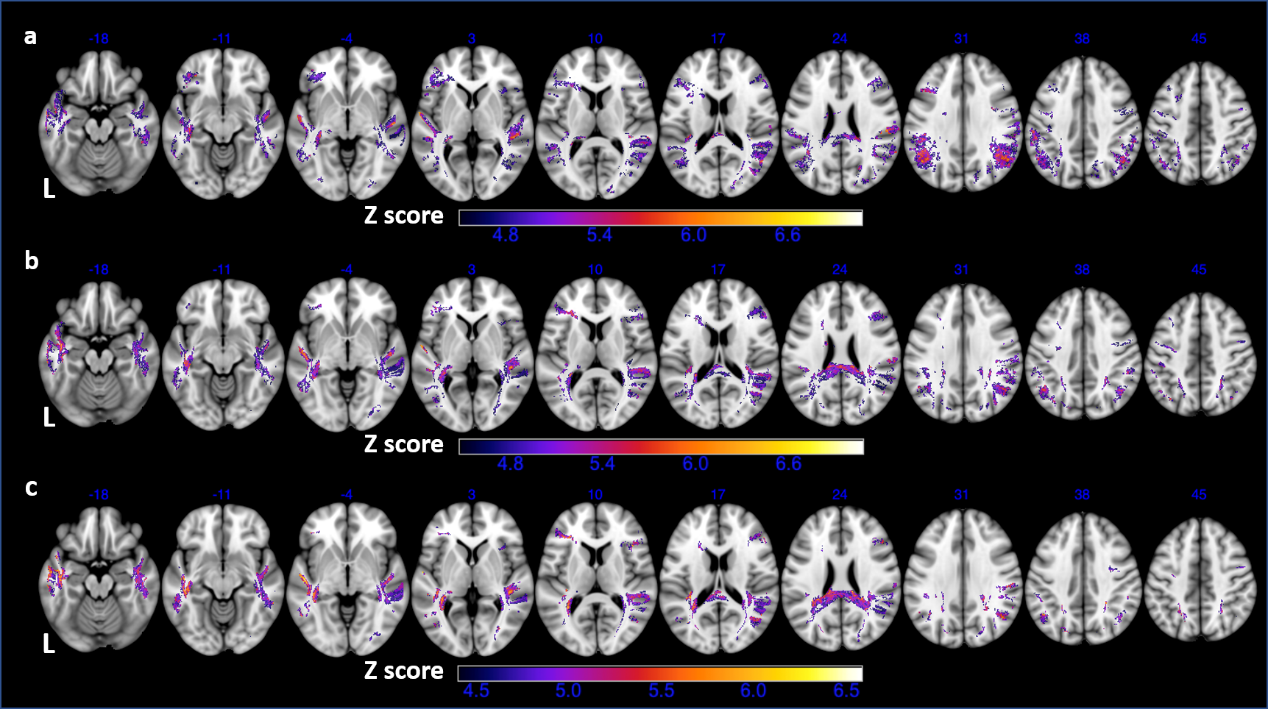


**Supplemental Figure III. Results of VDSM with SDC map binarized at different thresholds.** Voxel-wise disconnection severity thresholds were set at 1%, 10% and 20% for a, b and c, respectively. Axial coordinates refer to MNI space in mm. L indicates left.


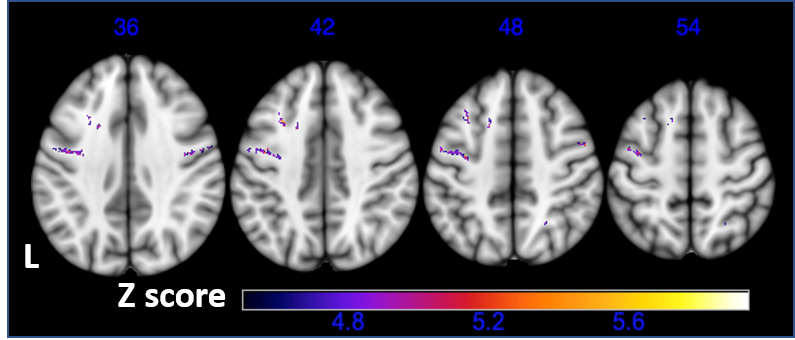


Supplemental Figure IV. Results of VDSM for ***Depressed mood***


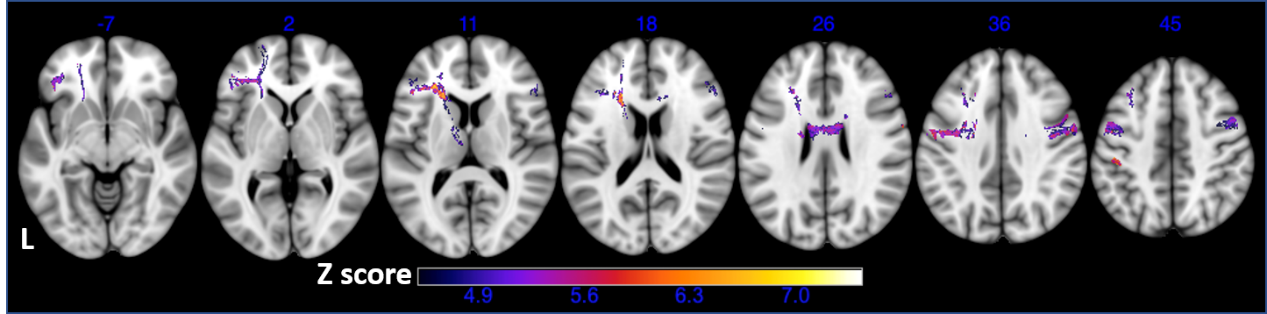


Supplemental Figure V. Results of VDSM for ***Retardation***


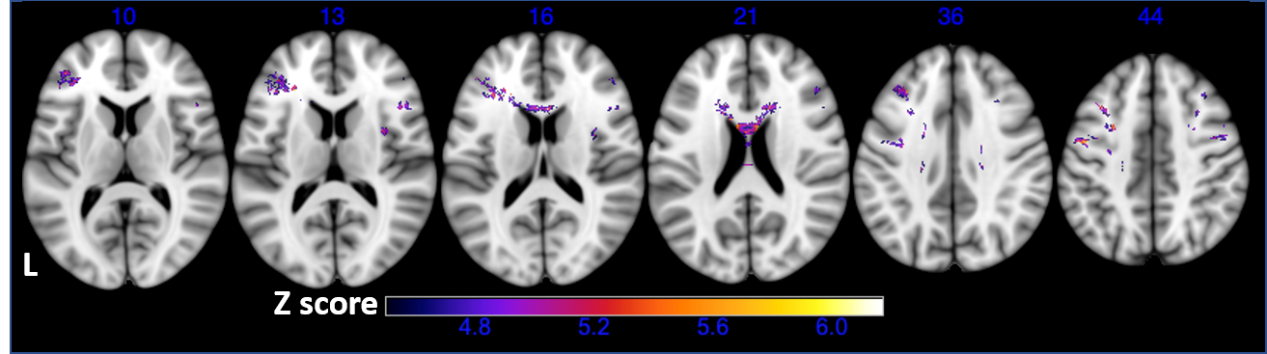


Supplemental Figure VI. Results of VDSM for ***General somatic symptoms***


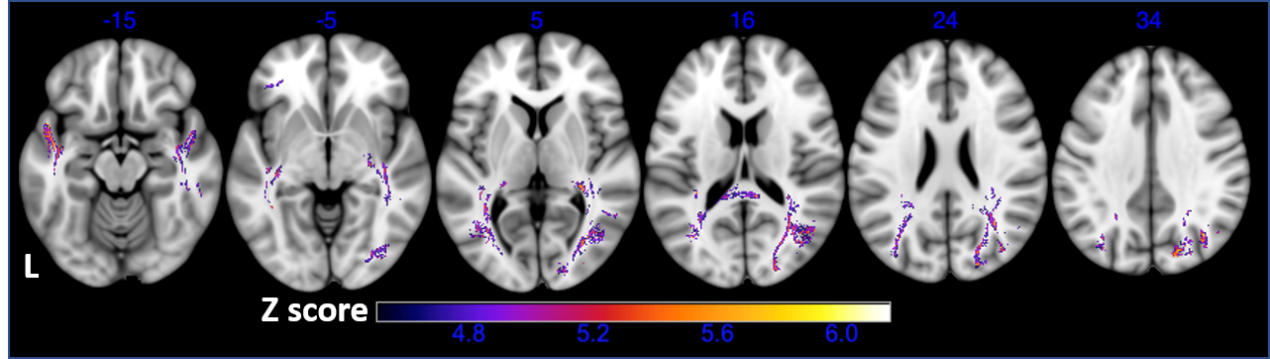


Supplemental Figure VII. Results of VDSM for ***Psychiatric anxiety***


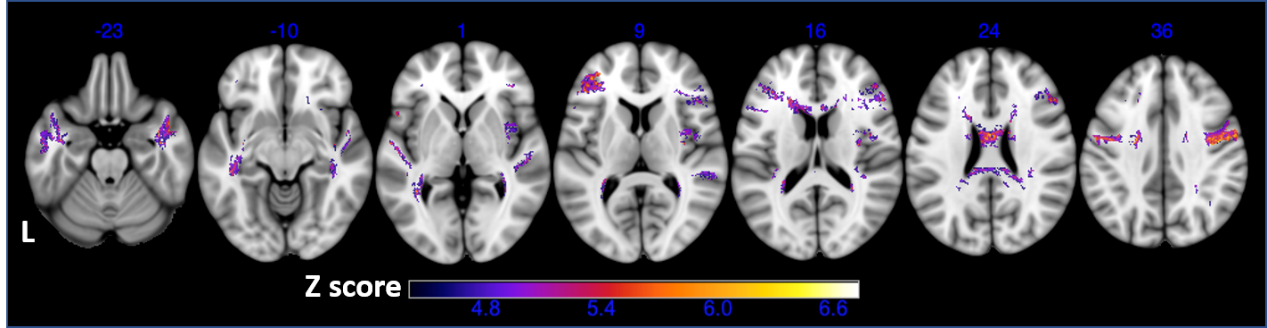


Supplemental Figure VIII. Results of VDSM for ***Loss of interest***

**References**

1. Yeh FC, Panesar S, Fernandes D, Meola A, Yoshino M, Fernandez-Miranda JC *et al*. Population-averaged atlas of the macroscale human structural connectome and its network topology. *Neuroimage* 2018; **178**:57-68.

2. Gozzi SA, Wood AG, Chen J, Vaddadi K, Phan TG. Imaging predictors of poststroke depression: methodological factors in voxel-based analysis. *BMJ Open* 2014; **4**:e004948.

3. Kim NY, Lee SC, Shin JC, Park JE, Kim YW. Voxel-based lesion symptom mapping analysis of depressive mood in patients with isolated cerebellar stroke: A pilot study. *Neuroimage Clin* 2017; **13**:39-45.

4. Padmanabhan JL, Cooke D, Joutsa J, Siddiqi SH, Ferguson M, Darby RR *et al*. A Human Depression Circuit Derived From Focal Brain Lesions. *Biol Psychiatry* 2019; **86**:749-758.

5. Klingbeil J, Brandt ML, Wawrzyniak M, Stockert A, Schneider HR, Baum P *et al*. Association of Lesion Location and Depressive Symptoms Poststroke. *Stroke* 2022:101161STROKEAHA122039068.

6. Grajny K, Pyata H, Spiegel K, Lacey EH, Xing S, Brophy C *et al*. Depression Symptoms in Chronic Left Hemisphere Stroke Are Related to Dorsolateral Prefrontal Cortex Damage. *J Neuropsychiatry Clin Neurosci* 2016; **28**:292-298.

7. Weaver NA, Lim JS, Schilderinck J, Biessels GJ, Kang Y, Kim BJ *et al*. Strategic infarct locations for post-stroke depressive symptoms: a lesion- and disconnection-symptom mapping study. *Biol Psychiatry Cogn Neurosci Neuroimaging* 2021.
